# Books and Authors

**Published:** 1911-06

**Authors:** 


**Diagnostic and Therapeutic Technic.** A Manual of Practical Procedures employed in Diagnosis and Treatment. By **Albert S. Morrow,** A.B., M.D., Adjunct Professor of Surgery in the New York Polyclinic. Octavo, pp. 775, with 815 illustrations, mostly original. Philadelphia and London: W. B. Saunders Co. 1911. (Cloth, $5.00 net.)

This book has apparently been written with a view to giving the general practitioner information which he will find of value in his everyday work. The illustrations in the main are well done and useful as descriptive of some of the results to be achieved. There are some, however, notably those showing views of the urethra through the endoscope, which are useless. They give no adequate idea of the pictures seen and should have been done from photographs by half-tone process.

In general the field of the different specialties has been invaded and yet so gentle the invasion and so intelligent the descriptions that one marvels at the author's adaptability. The book is marked by a display of commonsense and a clarity of description which is rather unusual in these books of such manifold interests. It has been written solely with a view to providing the physician with a description of the diagnostic and therapeutic methods in use by the best men in the profession.

In all cases descriptions of operations are omitted except in those rare instances where the operation must necessarily be considered one of emergency procedure, or where they form an important part of the diagnostic measures described. There is little or nothing left to the imagination of the reader. The careful and painstaking attention to detail is as remarkable as it is welcome. This is a work which will prove of great value.

**New and Nonofficial Remedies, 1911:** Containing descriptions of articles which have been accepted by the Council on Pharmacy and Chemistry of the American Medical Association, prior to January 1, 1911. Pp. 282. (Paper, 25 cents; cloth, 50 cents.)

This 1911 edition of the annual new and non-official remedies, issued by the council on pharmacy and chemistry of the American Medical Association, contains descriptions of all articles approved by the council up to December 31, 1910. There are also descriptions of a number of unofficial non-proprietary articles which the council deemed of value. The action, dosage, uses and tests of identity, purity and strength of articles are given.

Besides indicating to physicians the proprietary articles which the council's examination has found to be honestly marketed, and containing accurate descriptions of these articles, all similar articles are arranged under group headings; thus the physician at a glance can learn that atoxyl and soamin are practically identical articles, and that arsacetin is a closely related body. Again, the several proprietary solutions of the blood-pressure-raising principle of the supernatural gland are listed under a general title "epinephrin," and the manner in which the solutions differ from each other can be learned at a glance.

---

**Progressive Medicine,** March, 1911. A Quarterly Digest of advances, Discoveries and Improvements in the Medical and Surgical Sciences. Edited by **Hobart Amory Hare,** M.D., Professor of Therapeutics and Materia Medica in the Jefferson Medical College, Philadelphia, and **Leighton F. Appleman,** M.D., Instructor in Therapeutics in Jefferson Medical College. Philadelphia and New York: Lea & Febiger. (Per annum, paper bound, $6.00; cloth, $9.00.)

This volume is complete in every detail and covers the field of medicine and surgery thoroughly. Surgery of the head, neck and thorax is furnished by Charles H. Frazier; Infectious diseases, including acute rheumatism, croupous pneumonia and influenza, by John Ruhräh; Diseases of children, by Floyd M. Crandall; Rhinology and laryngology, by D. Braden Kyle; Otology by Arthur B. Duel.

---

**International Clinics.** A Quarterly of Illustrated Clinical Lectures and especially prepared articles on Treatment, Medicine, Surgery, Neurology, Pediatrics, Obstetrics, Gynecology, Orthopedics, Pathology, Dermatology, Ophthalmology, Otology, Rhinology, Laryngology, Hygiene, etc. Edited by **Henry W. Cattell,** M.D. Vol. I, twenty-first series. Philadelphia and London: J. B. Lippincott Co. 1910. (Cloth, $2.00.)

An especially valuable and interesting number is this of a particularly desirable series of publications. In the department devoted to diagnosis and treatment the spread of pellagra throughout the United States is considered by George A. Zeller. There is a further contribution on the treatment of syphilis with Ehrlich's dioxydiamidoarsenobenzol, by Wechselmann who, of course, is an authority on the subject. The value of blood pressure estimation is another important contribution. In the department of medicine the two most interesting and instructive articles are Syphilis of the circulatory system, by John H. Blackburn, and Recent clinical investigation of poliomyelitis, by Charles K. Mills.

In the department devoted to surgery C. P. Thomas writes on The open or surgical treatment of fractures; Ulceration of the male bladder is described by William Cullen Bryant and Carl Beck contributes articles on Symmetrical gangrene of both feet and Carcinoma of the thyroid.

Progress of medicine in 1910 in surgery, treatment and medicine is covered by complete reviews by J. C. Bloodgood, A. A. Stevens and John Musser.

---

**Hughes' Practice of Medicine.** Tenth edition. By **R. J. E. Scott,** M.A., B.C.L., M.D., Attending Physician to the Demilt Dispensary, New York; Author of "The State Board Examination Series." 12·mo, pp. 850. Philadelphia: P. Blakiston's Sons & Co. (Flexible leather, $2.50.)

This tenth edition of Hughes' valuable book has been very thoroughly revised. Many changes and additions were necessary and there has been some rearrangement of the subject matter in order to adopt it to modern classification. Several entirely new sections and numerous tables of differential diagnosis have been added, the whole resulting in an increase of about one hundred pages.

Assuming that the main efforts of the practitioner are to find out what is the matter with his patient and then to alleviate or cure, the sections on treatment are very complete and contain about 400 prescriptions. The inclusion of diseases of the skin, a subject generally omitted from works on practice, has gone far towards making the great popularity of the book. The same may be said in regard to the shorter article on mental diseases.

Dr. R. J. E. Scott, the editor, has had a wide literary and medical experience. He is well known by his contributions to the *New York Medical Record* on State Board Examinations, a subject with which he has become acquainted by reason of having made it a special study in connection with his teaching work and the preparation of the State Board Examination Series.

---

**Modern Diagnosis and Treatment of Diseases of Children.** By **Herman B. Sheffield,** M.D., Instructor in Diseases of Children at the New York Post-Graduate Medical School and Hospital. Octavo, pp. 619. Illustrated. Philadelphia: F. A. Davis Co. 1911. (Cloth, $4.50.)

As a clinical treatise of the medical and surgical diseases of infancy and childhood this book easily takes its place as one of the foremost works on the subject with which it deals. Based largely upon the author's personal experience and dealing with the essentials of the theory of pediatrics it presents much of the material contained within its covers in a manner which is new and entirely refreshing. There is noticeable lack of that prevalent tendency to give much space to quotations of various authors and a display of the fads and fancies of enterprising tradesmen, which means that there are none of those pages of commercial display of apparatus bearing the names of manufacturers so frequently found in medical textbooks.

The elimination of this space filling device gives the author of this book opportunity for the use of many photographs of conditions which he describes in his text and for the use of explanatory notes. However any individual reader may look upon the advisability of leaving out the apparatus pictures, which may be seen in almost every instrument catalogue, it is a distinct advance in book making and is welcome.

Physical diagnosis is given ample space and freely considered. There are special chapters on general and special therapeutics and the mental diseases of childhood. A system of side notes makes the book especially easy of reference. That portion devoted to the genital tract and diseases of the bladder is especially good and the treatment laid down in each case is eminently suitable for everyday practice. The illustrations are excellent and the book deserves success.

